# Silicone implant surface microtopography modulates inflammation and tissue repair in capsular fibrosis

**DOI:** 10.3389/fimmu.2024.1342895

**Published:** 2024-03-19

**Authors:** Ines Schoberleitner, Klaus Faserl, Christoph H. Tripp, Elisabeth Judith Pechriggl, Stephan Sigl, Andrea Brunner, Bettina Zelger, Natascha Hermann-Kleiter, Leoni Baier, Theresia Steinkellner, Bettina Sarg, Daniel Egle, Christine Brunner, Dolores Wolfram

**Affiliations:** ^1^ Department of Plastic, Reconstructive and Aesthetic Surgery, Medical University of Innsbruck, Innsbruck, Austria; ^2^ Protein Core Facility, Institute of Medical Chemistry, Biocenter, Medical University of Innsbruck, Innsbruck, Austria; ^3^ Department of Dermatology, Venereology and Allergology, Medical University of Innsbruck, Innsbruck, Austria; ^4^ Department of Anatomy, Histology and Embryology, Institute of Clinical and Functional Anatomy, Medical University of Innsbruck, Innsbruck, Austria; ^5^ Institute of Pathology, Neuropathology and Molecular Pathology, Medical University of Innsbruck, Innsbruck, Austria; ^6^ INNPATH GmbH, Tirol Kliniken, Innsbruck, Austria; ^7^ Institute of Cell Genetics, Department for Genetics and Pharmacology, Medical University of Innsbruck, Innsbruck, Austria; ^8^ Department of Obstetrics and Gynecology, Medical University of Innsbruck, Innsbruck, Austria

**Keywords:** SMI (silicone mammary implants), implant surface topography, FBR (foreign body response), implant encapsulation, pro-inflammatory mechanical stress mechanisms, SMI surface protein adsorption, immunomics, HSP60-mediated T-cell activation

## Abstract

Excessive fibrous capsule formation around silicone mammary implants (SMI) involves immune reactions to silicone. Capsular fibrosis, a common SMI complication linked to host responses, worsens with specific implant topographies. Our study with 10 patients investigated intra- and inter-individually, reduced surface roughness effects on disease progression, wound responses, chronic inflammation, and capsular composition. The results illuminate the significant impact of surface roughness on acute inflammatory responses, fibrinogen accumulation, and the subsequent fibrotic cascade. The reduction of surface roughness to an average roughness of 4 μm emerges as a promising approach for mitigating detrimental immune reactions, promoting healthy wound healing, and curbing excessive fibrosis. The identified proteins adhering to rougher surfaces shed light on potential mediators of pro-inflammatory and pro-fibrotic processes, further emphasizing the need for meticulous consideration of surface design. The composition of the implant capsule and the discovery of intracapsular HSP60 expression highlight the intricate web of stress responses and immune activation that can impact long-term tissue outcomes.

## 1 Introduction

Capsular fibrosis, also referred to as capsular contracture, stands as a well-documented complication associated with silicone breast implants, characterized by the formation of a fibrous capsule around the implant following the body’s natural response to foreign objects (1–4). This condition progressively leads to breast firmness, distortion, and intermittent discomfort, often requesting revision surgery (3, 4). A proposed hypothesis implicates an exaggerated immune reaction to antigens binding and/or modified by silicone as a potential etiological factor (5). In 2017, Efanov et al. and in 2014, Maijers et al. demonstrated diverse immune responses elicited by silicone breast implants, resulting in collagen production, constrictive capsule formation, and elevated levels of immune cells and cytokines in the bloodstream of affected women (6, 7). Subsequent research has revealed the activation and perpetuation of local immune responses in capsular tissue (8) through serum proteins, blood cells, and adjacent tissue cells, with specific serum proteins (fibronectin, IgG, CRP, HSP 60) (9) identified as central to innate and adaptive immunity (5, 10, 11). Patients exhibiting pronounced fibrotic reactions to silicone implants often exhibit elevated serum concentrations of these proteins on implant surfaces, potentially impacting immune responses and fibrosis development (11, 12). Utilizing advanced proteomic techniques, comprehensive protein profiles in the acute wound post-implantation have been identified, exposing inflammation and tissue turnover dysregulation, distinct from plasma, with observed time-dependent variations persisting over several months (13).

Limited data exist regarding localized immune responses and lymphocyte-related immune cell activity within fibrous capsules. In prior research we focused on fibrous capsules surrounding silicone breast implants (SMIs), revealing an immune response near the silicone surface, forming a “pseudo synovium” comprising macrophages, T-cells, and CD1a/CD208+ dendritic cells (14). Higher regulatory T cell (Tregs) levels inversely correlated with fibrosis severity (Baker scores I-IV) in peri-SMI capsules (15). Severe capsular contracture (Baker scores III-IV) exhibited reduced Tregs, which suppressed peripheral T effector cells, particularly during early fibrosis stages, by down-regulating TH1/TH17+ effector cells and limiting profibrotic cytokine production (15).

Capsular contracture’s precise cause remains elusive, but it likely involves intricate interactions between the immune system and implants. Studies on breast implant-associated anaplastic large cell lymphoma (BIA-ALCL) (16), a rare cancer linked to breast implants, have revealed unique immune cell compositions in patient capsules, hinting at the immune system’s role in its development (16, 17). Recent research underscores the host’s immune system’s crucial role in reacting to implant surface characteristics, particularly topography and roughness, which significantly impact immunoreactivity (18).

Implants can be classified by surface topography into smooth (Ra < 10 μm), microtextured (10 μm ≤ Ra ≤ 50 μm), or macrotextured (Ra > 50 μm) according to the International Organization for Standardization (ISO) (19). Clinical studies suggest that different surface architectures elicit distinct foreign body immune responses and fibrosis tendencies (18, 20–26). Higher surface texture complexity reduces the risk of implant malposition or rotation but is associated with a higher risk of biofilm formation and BIA-ALCL (27–29). *In vitro* studies revealed that silicone breast implant surface roughness influences the immune response, notably affecting cytokine profiles like IL-6, TGF-β, and TNF-α (18). This can potentially contribute to increased fibrosis. Additionally, *in vitro* analysis of monocyte/macrophage markers showed differential expression on various surfaces (18). *In vivo* studies in rat models and human tissue samples confirmed that specific surface textures can induce a pro-inflammatory immune response. Implants with a 4 µm average roughness resulted in the least inflammation and fibrous scar tissue (25). Our latest patient data corroborates improved biocompatibility with minor capsule formation around 4 µm roughness implants (26).

Our study addresses a crucial gap by comparing acute and chronic immune responses to differently textured silicone mammary implants (SMIs) in patients undergoing prophylactic nipple-sparing mastectomy (NSME) and SMI-based breast reconstruction. Specifically, we focus on silicone tissue-expanders with an average surface roughness of 4 µm, comparing them to those with a roughness of 60 µm Ra. According to the American Society of Plastic Surgeons (ASPS), the two-stage expander-based breast reconstruction, employing inflatable SMI, remains the prevailing choice (30). In this procedure, a partially inflated tissue expander is initially positioned, followed by a second stage conducted several months later, after pocket formation is complete, to replace it with a permanent implant. This two-stage approach serves to alleviate the initial pressure on the potentially compromised mastectomy skin flap. Additionally, it allows for the selection of the ideal permanent implant after a period of expansion and provides an opportunity to fine-tune the implant pocket during the second stage (31–33). Our comprehensive approach involves analyzing plasma (pre-op), wound bed fluid (1-5 days post-op), and tissue expander as well as capsular tissue (6-8 months post-op).

During surgery, we intraoperatively compare two types of tissue expanders: the CPX® 4 (termed from here on SMI 60 µm, roughness radius: 60µm Ra; Mentor) and the SmoothSilk® (termed from here on SMI 4 µm, roughness: 4µm Ra; Motiva), which differ in surface topography. To understand the immune response, we employ various techniques. These include a Tandem Mass Tag (TMT)-based quantitative proteomic approach to track protein expression dynamics in the acute post-op wound, flow cytometry to study immune cell activation in wound bed fluid samples, real-time qPCR and multiplex ELISA assays to analyze gene expression and cytokine secretion, and the assessment of chronic pro-inflammatory and fibrotic stimuli associated with early-stage fibrosis by stripping the device-associated proteome from the surface. We profiled intracapsular immune cell populations by flow cytometry as well as immunohistochemistry, and cytokine expression by real-time qPCR.

Our results demonstrate that SMIs with an average surface roughness of 4 µm mitigate acute inflammation and fibrosis, leading to reduced TH1/TH17 immune cell responses and pro-inflammatory signaling. Conversely, rougher SMIs induce T-cell response activators and chronic fibrosis drivers. Notably, smoother SMIs lead to thinner capsules with increased Treg activity and reduced HSP60 levels.

In summary, our immunomic study provides comprehensive insights into how the surface topography of SMIs impacts immune responses, emphasizing the potential for improved biocompatibility with smoother surfaces.

## 2 Materials and methods

### 2.1 Study population

**Figure 1:**
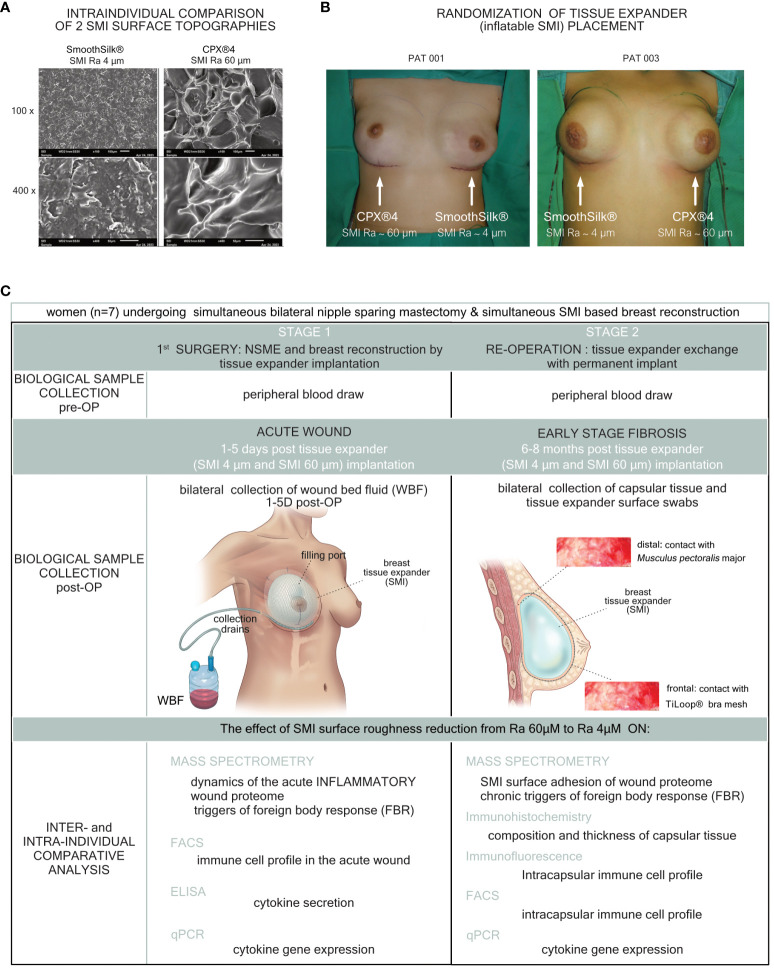
Standardized study design and intra-operative photo documentation of a bilateral tissue expander-based breast reconstruction. Each patient received both types of expanders, **(A)** the novel surface-roughness reduced SmoothSilk® breast expanders (Motiva Flora®, Establishment Labs, Costa Rica: surface roughness ∼ 4µm Ra; termed SMI 4 µm) and the routinely used CPX®4 breast expanders (MENTOR®, USA: surface roughness ∼ 60 µm Ra; termed SMI 60 µm), randomized to the left or right breast after bilateral prophylactic NSME. **(B)** Patient 003; Right: SMI 4 µm (SmoothSilk®), Left: SMI 60 µm (CPX®4), **(C)** Patient 001; Right: SMI 60 µm (CPX®4), Left: SMI 4 µm (SmoothSilk®).

As described previously in (13, 26), this study comprised 10 female patients who underwent simultaneous prophylactic bilateral nipple-sparing mastectomy (NSME) and tissue expander-based breast reconstruction (**Figure 1**). Written informed consent was obtained from all patients after confirming their eligibility based on the in-and exclusion criteria (**Table 1**). The consent covered photo documentation, the surgical procedure, sample collection, and the anonymized evaluation and publication of data. During the Expander-Immunology Trial, one patient withdrew due to the histological breast cancer diagnosis in on mastectomy sample, and two patients were excluded due to post-operative complications. As a result, seven patients were included in the evaluation. Patient demographic data and device information are summarized in **Table 2**.

**Table 1:** Inclusion and exclusion criteria for the Expander-Immunology trial. (ClinicalTrials.gov ID: NCT05648929).

	inclusion criteria		exclusion criteria
***1***	Female sex	***1***	Sever coagulation disorder, representing a potential contraindication for the elective surgery
***2***	Age > 18 years	***2***	Rheumatic disease accompanied by obligatory intake of immunomodulating therapeutic agents
***3***	High-risk family history for breast and/or ovarian cancer and/or BRCA1/2 gene mutation carrier	***3***	Severe renal functional disorder: renal insufficiency status IV or V (estimated glomerulary filtration rate (GFR) < 30ml/min)
***4***	Planned bilateral mastectomy with simultaneous breast reconstruction	***4***	Active hematological or oncological disease
***5***	Signed informed consent form.	***5***	HIV-Infection
		***6***	Hepatitis-Infection
		***7***	Pregnancy or breast-feeding
		***8***	Intake of anti-inflammatory drugs
		***9***	Carrier of silicone implants (e.g. gastric banding, mammary implants)

**Table 2:** Study population characteristics and device information.

Implantation site	Left: SMI 4 µm; Right: SMI 60 µm			Left: SMI 60 µm; Right: SMI 4 µm				
	PAT 001_001	PAT 001_002	PAT 001_007	PAT 001_003	PAT 001_004	PAT 001_005	PAT 001_006	
Vital parameters								
age (y)	34	41	30	31	26	60	33	
weight (kg)	86,3	54	108	54	58	105	57	
size (cm)	186,5	155,5	172	167	159	176	166	
body mass index	24.8	22.3	36.5	19.4	22.9	33.9	20.7	
body surface area	2.12	1.52	2.9	1.6	1.59	2.2	2.7	
Statust of natural breast								
asymmetry	no	no	no	no	no	no	no	
scars	no	no	no	no	no	no	no	
diseases	no	no	no	no	no	no	no	
active smoker	yes	no	no	no	no	no	no	
allergies	no	no	no	no	no	no	no	
Chronic diseases								
diabetes	no	yes	no	no	no	no	no	
Other								
job	manual job	office job	office job	office job	office job	manual job		office job
physical training (h/week)	>2	>2	0.5-2		>2	0.5-2		0.5-2
dominant hand	right	right	right	right	right	right		right
1st OPERATION: tissue expander implantation								
Bilateral prophylactic NSME resection weight [g]								
right breast	449	187	980	200	400	750	208	
left breast	471	167	1060	200	450	575	252	
Prepectoral reconstruction volume [cc]								
SMI 4 µm	450	260	570	260	440	570	260	
SMI 60 µm	440	250	550	250	450	550	250	
Intra-operative filling	250	150	550	150	300	500	150	

All donor biological samples (blood, wound bed fluid, capsular tissue, and removed tissue expander) and information were obtained with the informed written consent of the participants and in accordance with: (i) the regulations of the relevant clinical research ethics committee as well as (ii) the Declaration of Helsinki and with (iii) The European Union Medical Device Regulation (§ 40 section 3 Medical Devices Act). Therefore, 14 peripheral blood draws (7 patients x 2 surgeries), 70 WBF samples (7 patients x 5 days post-op x 2 tissue expander types), 28 capsular tissue specimens (7 patients x 5 days post-op x 2 tissue expander types x 2 sampling locations) and 14 tissue expander surface strips (7 patients x 2 expanders) were evaluated. For a detailed description of the study population see Schoberleitner et al., 2023 (13, 26).

### 2.2 Study design

This monocentric, randomized, double-blind controlled clinical study was approved by the Institutional Ethical Committee of the Medical University Innsbruck, Austria (approval no. 1325/2019, 23 January 2020, session 405) and the Austrian Federal Office for Safety in Health Care (approval no. 13340962). To analyze inter- and intra-individually a universal (directed to all SMI with same shell composition) immune reaction and to analyze an SMI shell surface topography-dependent SMI-associated host response, we chose to implant two tissue expanders, both composed of a poly(dimethyl siloxane) (“PDMS”) elastomer shell, with varying surface topographies. We evaluated a total of 7 patients, who received either the routinely used expander Mentor CPX™4 (termed SMI 60 µm) or the novel Motiva SmoothSilk® with reduced surface topography roughness (termed SMI 4 µm), randomized to left or right breast after prophylactic bilateral NSME (**Figure 1**). The patient and laboratory expert were double-blinded. Matching was performed intra-individual and conducted according to the implanted tissue expander. The inflatable tissue expanders were exchanged for definite implants in a second surgery, 6 to 8 months post-implantation. For detailed description of the study design see Schoberleitner et al., 2023 (13, 26).

### 2.3 Biological sample collection

The blood draws were conducted concurrently with anesthesia before both the initial tissue-expander implantation and the subsequent tissue-expander removal/exchange with a definitive implant. Biological samples of wound bed fluid (referred to as WBF) were collected daily from day 1 to 5 following expander implantation. Wound drains, integral to the surgical procedure for patients undergoing expander-based reconstruction, were retained postoperatively. WBF was collected under sterile conditions in sterile containers at room temperature. For the initial 24 hours, no vacuum was applied to the drains. However, after this period, the drains were maintained with a vacuum until removal. Flasks containing WBF were removed every 24 hours within the timeframe of 24 to 120 hours postoperatively, representing the total collection time of 120 hours. Subsequently, the collected WBF was transported to our research laboratory for cell culture. For immune profiling through qPCR, portions of the samples were aseptically frozen at -80°C. To isolate proteinaceous and cellular fractions from peripheral blood and WBF, we employed gradient separation of drain fluid using Ficoll-Paque® (Cytivia) to eliminate the cellular component. The cellular fraction was then subjected to further processing for immune profiling analysis via flow cytometry. The proteinaceous WBF was subsequently sterilized by passing it through a 0.1µm and then a 0.07µm syringe filter to remove all cells, both human and microbial. The resulting proteinaceous fraction was frozen at -80°C for subsequent processing using a TMT-based quantitative proteomic approach and Immunoassays.

During reoperation, capsular tissue (3 × 3 cm) was harvested from both implants, at 2 positions, anterior contact zone with TiLOOP® and posterior (TiLOOP® free) contact zone with *M. pectoralis*. Samples were placed immediately after withdrawal into sterile boxes stored at 4°C before transport to the research laboratory. Under sterile conditions, the specimens were divided for three types of analysis: (i) For immunohistochemistry and immunofluorescence analysis, they were fixed in formalin and subsequently processed at the Institute of Pathology, Neuropathology, and Molecular Pathology and the Department of Anatomy, Histology, and Embryology, Division of Clinical and Functional Anatomy. For immune profiling by flow cytometry (ii) and qPCR(iii), they were frozen at -80°C.

Expander exchange with definite implants was performed during re-operation between 6 to 8 months after initial expander implantation. Removed tissue expanders were placed immediately after withdrawal into sterile boxes and frozen as well as stored at -80°C before transport to the research laboratory of the Protein Core Facility, for label-free quantitative proteomic analysis.

### 2.4 The mass spectrometry proteomics data source

For detailed description of biological sample preparation, TMT-based quantitative proteomic approach, and label-free quantitative proteomic analysis see Schoberleitner et al., 2023 (13). The mass spectrometry proteomics data from plasma, wound bed fluid specimens, and adhesive SMI proteome specimens (13); ProteomeXchange Consortium via the PRIDE partner repository with the dataset identifier PXD039840, were subjected to:

*a. Identification, Characterization, and Quantification of differential common and topography-exclusive wound bed proteome*

Obtained data from plasma and wound bed fluid specimens were log2 transformed and analyzed for common and exclusive to SMI 4 µm or SMI 60 µm set of proteins associated with both devices in the acute wound as well as interaction with the plasma proteome by Interactivenn (34).

Identified common proteins to both devices were tested for Pearson r correlation of log2 protein abundance (common plasma- and local tissue-derived WBF samples over time). Identified SMI surface topography-exclusive proteins were tested for enriched Gene Ontology (biological process, cellular compartment, molecular function) as well as Kyoto Encyclopedia of Genes and Genomes (KEGG) pathway terms by Shiny GO software (version 0.77) (35). Significance was tested with a two-sample test with a false discovery rate according to Benjamini-Hochberg set to 0.05. The Search Tool for the Retrieval of Interacting Genes/Proteins (STRING v 11.5) database of physical and functional interactions was used to analyze the protein-protein interaction (PPI) of selected proteins. Pearson r correlation of log2 protein abundance was applied to test plasma- and local tissue-derived WBF samples over time.

Statistical data analysis of the common and topography-exclusive wound proteome was carried out with GraphPad Prism (version 9.4.1). Mean values and standard deviations were calculated for each experimental condition or type of sample. p-values between samples were calculated by unpaired t-test per protein, with individual variances computed for each comparison, combined with the two-stage linear step-up procedure of Benjamini, Krieger, and Yekutieli. Significance was tested with a two-stage set-up method with a false discovery rate set to 0.01. Proteins were regarded as being differentially expressed when meeting the criteria l2fc ≥ ± 1.5 and adjusted p-value ≤ 0.01. Heatmaps were generated using the ClustVis (36) tool. Generation of tables was performed with Microsoft Excel 2018 (Microsoft Corporation). Generation of correlation plots was performed using GraphPad Prism (version 9.4.1).

*b. Identification and Characterization of common adsorbed wound bed proteome on SMI surface*

Obtained abundances from adhesive SMI proteome specimens were analyzed for a common set of proteins adsorbed to both devices by Interactivenn (34). Identified SMI topography-exclusive adsorbed proteins were submitted to Gene Ontology (biological process, cellular compartment, molecular function) by Shiny GO software (version 0.77) (35). Functional categories with an adjusted p-value < 0.05 (Benjamini-Hochberg) were defined as significantly enriched. Heatmaps were generated using the ClustVis (36) tool. Generation of tables was performed with Microsoft Excel 2018 (Microsoft Corporation). Generation of correlation plots was performed using GraphPad Prism (version 9.4.1).

### 2.5 Scanning electron microscopy

Implant morphology and topography were studied by scanning electron microscopy (SEM). Samples were prepared using the same procedure and concentration. They were then washed three times in PBS. Next, the patches went through a sequence of increasing alcohol concentrations, beginning with 50%, then 70%, 80%, and eventually 99% ethanol. After air-drying for a minimum of 5 minutes, the patches were attached to SEM pins and sputtered with gold using an AGAR sputter coater (P5240-012) for approximately one minute at 30 mA. Representative images of each surface were taken using a JSM-6010LV scanning electron microscope, Jeol GmbH, Freising, Germany.

### 2.6 FACS analysis

PBMC were obtained from EDTA preserved venous blood samples (15-60 ml blood), MNCs from collected wound bed fluid (10-75 ml) and total cell isolates from capsular fibrotic tissue specimens of study participants. PBMC and MNC were isolated using Ficoll-Paque™ (VWR) standard density gradient centrifugation method. As previously described (37), single-cell suspensions of freshly excised capsules were prepared using a mechanic sample separation along with enzymatic digestion by collagenase (Collagenase D; Roche, 110889000000) with termination through the addition of 20 ml sterile FBS. Sample suspensions were prepared in DMEM +10% FBS media (Gibco, A419201) and passed through 70 µm cell strainers (Falcon, REF 652350) to remove debris. All tissue-derived samples were also subjected to red blood cell lysis with 5 ml of 1× RBC lysis buffer (Biolegend, 420302) for 5 min at 4°C, with termination through the addition of 20 ml sterile PBS. Remaining cells were centrifuged at 300–400 g and 4°C and 10^6^ cells were resuspended in 245 μl of staining buffer (Biolegend, 420201) for staining in the dark for 30 min at 4°C. Surface stains included Biolegend monoclonal antibodies anti CD8-BV510 (clone SK1, cat no. 344732, 1:100), CD183-AF700 (clone G025H7, cat no. 353742, 1:50), CD196-BV605 (clone G034E3, cat no. 353420, 1:100), CD45RA-FITC (clone HI100, cat no. 304106, 1:50), CD197-PE (clone G043H7, cat no. 353204, 1:200), CD25-APC (clone BC96, cat. no 302609, 1:50), CD3-PE/Cy7 (clone OKT3, cat no. 317334, 1:100), CD4-PE/Cy5 (clone rpa-t4, cat no. 300510, 1:200) and ebioscience antibodies anti-CD45-APC-eF780 (clone h130, cat no. 47-0459-42, 1:100), CD25-APC (clone bc96, cat no. 17-0259-42, 1:50) as well as viability dye-BV450 (cat no. 65-0863-14). For intracellular FOXP3 Treg stains, ebioscience antibody FOXP3-PE (clone 236a/e7, cat no. 12-4777-42, 1:50) was applied. Following all washes, samples were resuspended in 500 μl of IOTest 3 1x fixation solution (Beckman Coulter, cat no. A07800) for eventual FACS analysis using a CytoFLEX (Beckman Coulter). Unstained, single-antibody controls were applied.

### 2.7 Reverse transcription real-time PCR

For RT-qPCR analyses blood, WBF, and capsular tissue as indicated in **Figure 1** were used. Briefly, all biological samples were frozen in triplicates at -80°C immediately after collection and processed for further analysis. The frozen blood and WBF samples were subjected to further extraction directly and the frozen tissues were pulverized by Covaris CryoPREP® Dry Impactor. Total RNA was extracted using TRI Reagent® (Sigma Aldrich) followed by RNA purification with Monarch RNA Clean up Kit (NEB) and cDNA synthesis with LunaScript RT SuperMix Kit (NEB). qPCR was performed in triplicate using Luna® Universal qPCR Master Mix (NEB) with 25 ng cDNA and 0.4 μM of target-specific primers in a Biorad CFX instrument (Biorad). Primer sequences are available upon request. Transcripts were normalized to *MT-ATP6* and *B2M* as previously described in (38). Proteobacterial *HSP60* (*GroEL*; NCBI Ref WP_00729117.1) was used as a negative control for *HSPD1* (NCBI Ref NM_199449.2) gene expression. 2^−ΔΔCt^ values were calculated and statistical analysis was done by unpaired Student’s t test (Graphpad Prism 8.2.1).

### 2.8 Multiplex immunoassay

The proteinaceous WBF fractions were subjected to cytokine level were quantification using Th1/Th2/Th9/Th17 Cytokine 18-Plex Human ProcartaPlex™ Panel and Immunoassay Kit according to manufacturer’s instructions (Invitrogen, Cat No. EPX110-10810-901). The assay enables the detection of the following cytokines: GM-CSF, IFN gamma, IL-1 beta, IL-2, IL-4, IL-5, IL-6, IL-12p70, IL-13, IL-18, TNF alpha, IL-9, IL-10, IL-17A (CTLA-8), IL-21, IL-22, IL-23, IL-27. The plate was run on a Bio-Plex 200 Systems instrument using Bio-Plex Manager 6.2 software (Biorad).

### 2.9 Immunohistochemistry

During reoperation, capsular tissue (3 × 3 cm) was harvested from both implants, at 2 positions, anterior contact zone with TiLOOP® and posterior (TiLOOP® free) contact zone with *M. pectoralis*. Samples of 7 patients were fixed by immersion in 4% paraformaldehyde in phosphate‐buffered saline (PBS, pH 7.4) for 24 hours followed by rinsing in PBS and routinely processed at the Institute of Pathology, Neuropathology and Molecular Pathology. Thus, for every patient, at least four specimens were evaluated with seven different antibodies. Immunohistochemistry was performed on an automated platform (Benchmark, ULTRA, Ventana Medical Systems, Tuscon, US). After de-paraffinization, slides were heat pre-treated with cell conditioning reagent 1 (CC1, Ventana Medical Systems, Tucson, US) for 30 (CD4, CD20, CD25) or 60 min at 95°C for antigen retrieval and incubated for 32 or 20 (CD8, CD20) min at 37°C with the primary antibody. For visualization, ultraView DAB Detection Kit (Ventana Medical Systems, Tucson, US) was used according to the manufacturer’s recommendation. Finally, slides were washed in distilled water, counterstained with Hematoxylin (12 minutes) and Bluing Reagent (4 minutes), dehydrated in descending order of alcohols, and cleared in xylene. Slides designated for immunohistochemistry profiling of immune cells were cover-slipped with Tissue-Tek (Sakura Finetek, Japan) mounting medium, and slides intended for HSP60 staining, with Entellan (Merck, Darmstadt, Germany) mounting medium. Immune cell profiling and HSP60 stains included murine monoclonal antibodies listed in **Table 3**.

**Table 3:** Primary murine monoclonal antibodies used in immunohistochemical stains.

Antibody*	Clone	Company	Dilution	Cell type	Staining pattern
Immunoprofiling					
CD3	2GV6	Ventana Medical Systems	pre-diluted	T-cells	membranous
CD4	SP35	Ventana Medical Systems	pre-diluted	T-helper cells	membranous
CD8	SP57	Ventana Medical Systems	pre-diluted	cytotoxic T-cells	membranous
CD25	4C9	Ventana Medical Systems	pre-diluted	regulatory T-cells	membranous
FOXP3	EP340	MEDAC/BSB	1:100	regulatory T-cells	nuclear
CD20	L26	Ventana Medical Systems	pre-diluted	B-cells	membranous
CD68	KP-1	Ventana Medical Systems	pre-diluted	macrophages	membranous
HSP60					
GroEL (bacterial HSP60)	A57-B9	Alexis Corporation	1:500	*Chlamydia trachomatis* HSP60 (A57-B9)	membranous
HSP60	611959	BD Transduction Laboratories ™	1:600	human HSP60	membranous

*All antibodies are CE-IVD certified.

### 2.10 Digital image analysis

Immunohistochemically stained slides were reviewed by two experienced pathologists to assess the morphology of inflammation and the quality of staining before digital image analysis.

After staining, whole slides were scanned using a high–performance Scanner (Pannoramic scanner, 3DHistech, Budapest, Hungary), and the scans were directly uploaded into the software (Qupath 0.3.2, open source software) used for digital image analysis based upon a machine learning approach. In brief, whole slides were annotated and a single threshold classifier was trained using a supervised machine learning classifier (random trees). As a training set five slides stained with CD3 and five slides stained with CD68 were selected to develop a classifier able to detect all immune cells.

Digital images of immunostained slices were acquired in AxioVision microscope software linked to an AxioCamHRc color camera and an AxioPlan 2 microscope (Zeiss, Jena, Germany). The immunohistochemical staining pattern was examined particularly for the mesenchymal surroundings.

### 2.11 Statistics

Graphing and statistics were performed using GraphPad Prism software v.8.2.1. The statistical details of experiments are presented in the relevant figure legends. The level for statistical significance was set at p ≤ 0.05 for all statistical tests and significant differences were marked (*p ≤ 0.05, **p ≤ 0.01, ***p ≤ 0.001, ****p ≤ 0.0001, ns, not significant).

## 3 Results

### 3.1 Surface roughness reduction slows pro-inflammatory response in the acute wound

**Figure 2:**
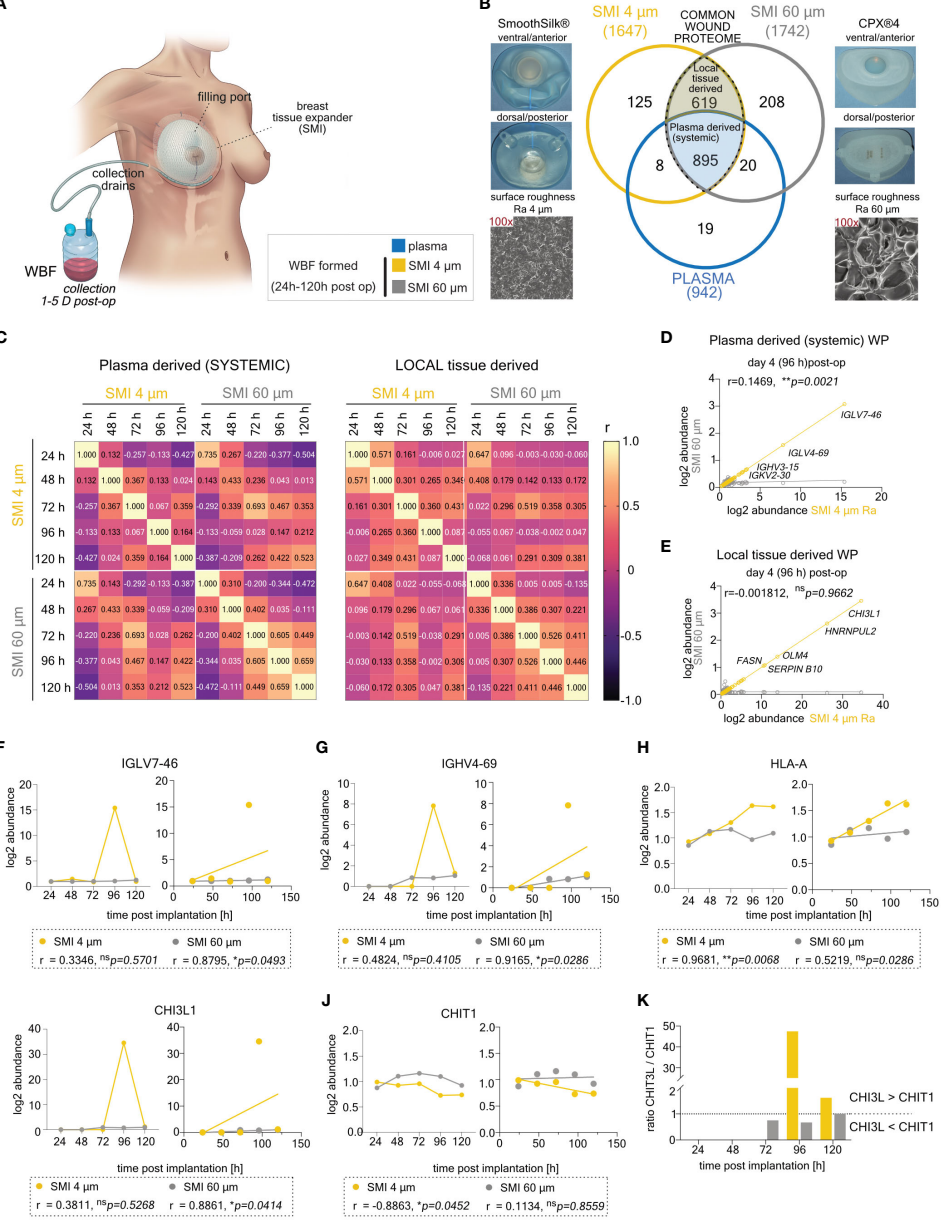
Differential expression of common plasma- and local tissue-derived WBF proteome 96 h post-implantation of SMI 4 µm and SMI 60 µm. **(A)** Schematic representation of WBF sample collection 24 – 120 h post-op. **(B)** Photo and SEM image of SMI 4 µm and SMI 60 µm and Venn diagram showing the distribution of proteins in the collected plasma (pre-op) and WBF formed around both SMI (24 – 120 h post-op; n=7). Shaded blue panel: common plasma-derived wound proteome found in plasma and WBF around both devices. Shaded yellow panel: common local tissue-derived wound proteome found in WBF around both devices but NOT in plasma. **(C)** Heatmap of correlation matrix showing Pearson r correlation of log2 protein abundance in common plasma- and local tissue-derived WBF samples over time. The r value for every pair was denoted in corresponding square units. **(D, E)** Scatter plot with regression line of timepoint dependent Pearson r (SMI 4 µm *vs*. SMI 60 µm) in **(D)** plasma- and **(E)** local tissue-derived WP abundance. Simple linear regression for protein abundance over time around both devices was calculated. Pearson r and p-value were denoted in the graph. **(F–J)** Comparative analysis of plasma-derived **(F)** IGLV7-46, **(G)** IGHV4-69, **(H)** HLA-A, and local tissue-derived **(I)** CHI3L, **(J)** CHIT1 log2 abundances (Left; dots with connecting lines, median shown) with calculated Pearson r of protein abundance x time point post-op (Right; scatterplot with simple regression line): in WBF formed around both SMI 24 – 120 h post-implantation. Pearson r and p-value for both SMI were denoted above the corresponding panel. **(K)** Time-course log2 abundance ratio of CHI3L/CHIT1 accumulated around both SMI (24 – 120 h post-op). The level for statistical significance was set at ^ns^p>0.05, *p<0.05, **p<0.002, ***p<0.0002, and ****p<0.0001, for all statistical tests (inter- and intra-individual comparison; n=7).

To explore the effects of reducing implant surface roughness from Ra 60 µm (SMI 60 µm; CPX®4, Mentor) to Ra 4 µm (SMI 4 µm; SmoothSilk®, Motiva) on inflammatory tissue repair post-implantation, we turned to our previously generated mass spectrometry profiles of the collected wound proteome samples (**Figure 2A**) (39). This dataset covered plasma and acute wound proteome profiles from both SMI types within 1 to 5 days post-operation, allowing us to compare protein distributions.

The acute wound proteome displayed a complex composition (**Figure 2B**), consisting of plasma-derived (**Supplementary Table S1**) and locally (**Supplementary Table S2**) differentially expressed proteins (DEPs) common to both SMI types or unique to differing surface roughness. Analyzing 895 common plasma-derived and 619 common local wound proteins, we conducted sample correlation analysis to uncover relationships between SMI 4 µm and SMI 60 µm proteomes, as well as roughness-exclusive protein accumulation over time.

Results revealed a significant inverse correlation between common proteomes and time (**Figure 2C**). Reduction of implant surface roughness disrupted the correlation of systemic (**Figure 2D**) and locally derived (**Figure 2E**) wound protein abundance with time on day 4 post-op within comparative WBF samples around both devices (SMI 60 µm *vs*. SMI 4 µm). Notably, around SMI 4 µm, there was an elevated systemic abundance of IGLV (**Figure 2F**) and IGHV (**Figure 2G**), indicating B-cell activation and response. A heightened MHC class I response was evident through significantly increased accumulation of HLA-A (**Figure 2H**) and HLA-C (**Supplementary Table S2**).

The plasma-derived acute proteome, accumulating around both devices, demonstrated SMI-induced hyperinflammatory responses, with complement activation, cytokine surge, coagulopathy, and ECM turnover (**Supplementary Figure S1A**). Immunity-associated proteins correlated to immune cell activation and regulation showed no quantitative difference between devices (**Supplementary Figure S1B**). The impact of roughness reduction on the plasma-derived proteome/systemic inflammatory reaction was marked by a significant decrease in IFIT3 (**Supplementary Figure S1C**), elevated M1 macrophage activation-associated CHIT3 (**Figures 2I, K**), and higher accumulation of fibrogenesis-associated proteins HSPA2 and FGA around the smoother device (**Supplementary Figure S1D**; SMI 4 µm). No differences were observed in ECM turnover-associated protein abundance.

COL I/III and TIMP/MMP equilibrium was notably disrupted around the rougher device (**Supplementary Figures S1F, H**; SMI 60 µm), leading to higher levels of TIMP2 and COL III post-operation (**Supplementary Figures S1E, G**). The profibrotic marker family S100A showed delayed activation around SMI 4 µm (**Supplementary Figures S1I, J**). Locally derived wound proteome reflected inflammatory signals (**Supplementary Figure S2A**), including an immediate IFIT2 spike (**Supplementary Figure S2B**; 24 h post-op), lower TGFβ (**Supplementary Figure S2C**), higher TNFα (**Supplementary Figure S2D**), and lower NFκB (**Figure 2E**) responses around SMI 4 µm.

Comparing protein abundance in plasma-derived systemic reaction and local acute wound indicated a shifted and decelerated proinflammatory response and chronic inflammation mediation around the smoother SMI 4 µm within the initial five days post-implantation.

### 3.2 Diminished implant surface roughness alters acute wound tissue repair mechanisms

**Figure 3:**
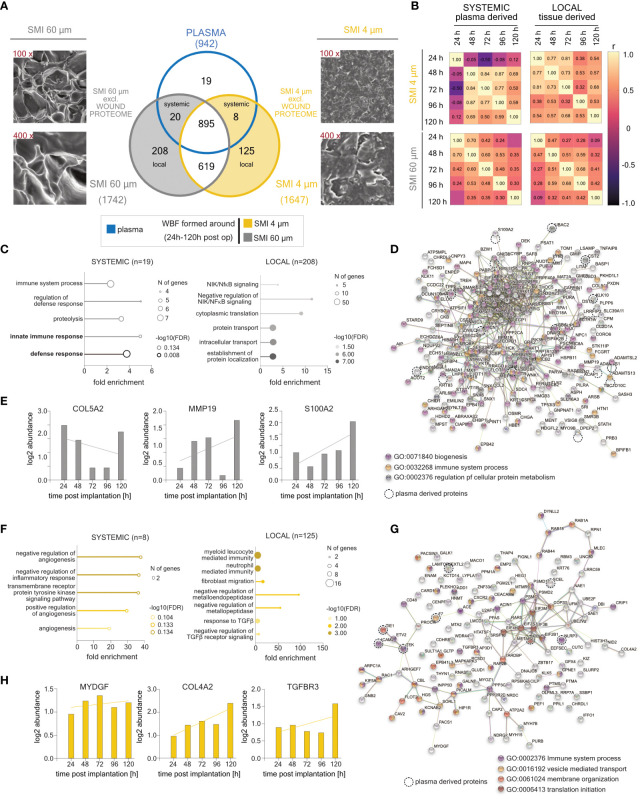
SMI surface topography-exclusive plasma- and local tissue-derived wound bed fluid proteome formed intra-individually around SMI 4 µm and SMI 60 µm post-implantation (24 – 120 h post-op; n=7). **(A)** SEM image of SMI 4 µm and SMI 60 µm surface (magnitude: 100x and 400x) and Venn diagram showing the distribution of proteins in the collected plasma (pre-op) and WBF formed intraindividual around both tissue expanders (24 – 120 h post-op). Shaded grey panel: SMI 60 µm Ra exclusive plasma- and local tissue-derived wound proteome found in plasma and WBF around both devices. Shaded yellow panel: SMI 4 µm Ra exclusive plasma- and local tissue-derived wound proteome found in WBF around both devices but NOT in plasma. **(B)** Heatmap of correlation matrix showing Pearson r correlation of log2 protein abundance in SMI surface topography-exclusive plasma- and local tissue-derived WBF samples over time. The r value for every pair was denoted in corresponding square units. GO biological process enrichment of systemic and local **(C)** SMI 60 µm and **(F)** SMI 4 µm exclusive wound proteome. Protein-protein interaction regulatory network based on STRING database of **(D)** SMI 60 µm and **(G)** SMI 4 µm exclusive wound proteome. Time course-dependent protein abundance of fibrosis markers enriched exclusively around **(E)** SMI 60 µm or **(G)** SMI 4 µm. dashed circle: plasma-derived proteins The level for statistical significance was set at ^ns^p>0.05, for all statistical tests (inter- and intra-individual comparison; n=7).

The acute wound proteome exclusive to SMI 4 µm comprised 8 plasma-derived (**Supplementary Table S3**) and 125 local tissue-specific (**Supplementary Table S5**) differentially expressed proteins (DEPs) (**Figure 3A**; **Supplementary Figure S3**). Conversely, 20 systemic (**Supplementary Table S4**) and 200 local (**Supplementary Table S6**) wound proteins were exclusive to SMI 60 µm (**Figure 3A**; **Supplementary Figure S4**). Both outcomes affirm that the local tissue exhibits a predominant immunomodulatory response to SMI topography. Sample correlation analysis confirmed regression of systemic and local protein abundances within the first five days in both surface-exclusive proteomes (**Figure 3B**).

For deeper insights into the functions of identified surface-exclusive wound proteins, we conducted Gene Ontology (GO) biological process enrichment analysis (**Figures 3C, F**) and a protein-protein interaction (PPI) regulatory network based on STRING database analysis (**Figures 3D, G**) for systemic and local SMI 60 µm and SMI 4 µm exclusive wound proteomes. GO analysis of the SMI 60 µm exclusive proteome indicated associations of plasma-derived fraction proteins with inflammatory and immune cell activating biological processes (neutrophil-mediated immunity), while the local wound proteome correlated with proinflammatory signals (NFκB signaling regulation) and protein signal transport (**Figure 3C**). The STRING analysis generated a PPI network with 227 nodes and 633 edges, an average node degree of 5.58, a local clustering coefficient of 0.42, an expected edge number of 529, and PPI enrichment p-value of 5.6e-06 (**Figure 3D**).

Conversely, GO analysis of the SMI 4 µm exclusive proteome indicated systemic proteins mainly involved in angiogenesis and negative regulation of inflammatory response, while the local wound proteome demonstrated mechanisms of anti-inflammatory tissue repair like fibrogenesis and negative regulation of metallo(endo) peptidases and TGFβ signals (**Figure 3F**). The STRING analysis resulted in a PPI network of 135 nodes and 134 edges, average node degree of 1.99, local clustering coefficient of 0.379, expected edge number of 105, and PPI enrichment p-value of 0.00333 (**Figure 3G**). In terms of the chronic progression from acute wound to chronic inflammation, our findings indicate selective accumulation of molecular fibrosis drivers (COL5A2, MMP19, S100A2) exclusively around the rougher device (**Figure 3E**), and recruitment of anti-angiogenic COL4A2 (40, 41) as well as anti-fibrotic mediators MYDGF (42, 43) and TGFBR3 (44, 45) to SMI 4µm post-op wound (**Figure 3H**). Notably, COL5A2 and MMP19 were strongly reduced 96h post-implantation, with a subsequent accumulation peak 120h post-op. Inflammatory markers LITAF (LPS-induced TNF-alpha factor) and LY6D (Lymphocyte antigen 6 family member D) were exclusively accumulated in WBF around the rougher SMI 60µm (**Supplementary Figure S4**).

Collectively, our data highlights a decelerated, differentiated, and reduced pro-inflammatory and -fibrotic foreign body response due to SMI surface roughness reduction within the acute wound 1-5 days post-implantation.

### 3.3 Enhanced proinflammatory and profibrotic-associated immune cell response due to rougher implant surface

**Figure 4:**
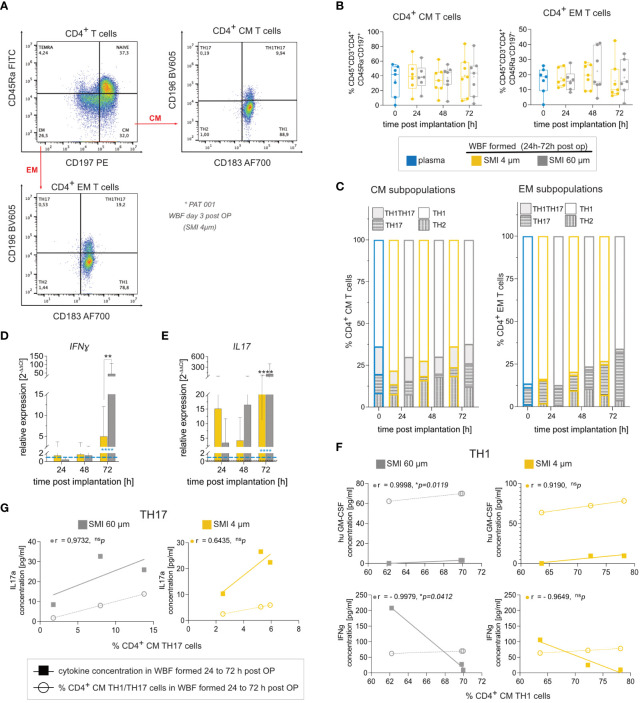
SMI surface roughness reduction effect on WBF immune cell populations and inflammatory response in the acute wound 24 – 72 h post-op. **(A)** Flow cytometry revealed a predominant response of **(B)** CM (CD45^+^CD3^+^CD4^+^CD45Ra^-^CD197^+^) and EM (CD45^+^CD3^+^CD4^+^CD45Ra^-^CD197^+^), **(C)** TH1 (CD183^+^ CD196^-^) cells and TH1TH17 (CD183^+^ CD196^+^) cells to both SMI surface topographies. CD4^+^ T cells from WBF surrounding SMI 60 µm expressed **(D)** interferon-gamma (IFNγ) and **(E)** IL17 (IL17A) at significantly higher levels 72 h post-op compared to WBF formed around SMI 4 µm. 2-way ANOVA of **(D, E)**: interaction (topography x time-point post-op) main effect: **(D)** F (4, 52) = 3.160, *p = 0.0213; **(E)** F (4,52) = 18.87, ****p<0.0001; topography main effect: **(D)** F(2, 52) = 2.97, ^ns^p=0.06; **(E)** F (2, 52) = 26.72, ****p<0.0001; time-point post-op main effect: **(D)** F(2, 52) = 3.953, *p = 0.0213; **(E)** F (2, 52) = 31.08, ****p<0.0001. Tukey´s multiple comparison test revealed time-point post-op main effect at 72 h: **(D)** ***p [plasma *vs.* WBF SMI 60 µm] =0.0007; **p [WBF SMI 4 µm *vs*. WBF SMI 60 µm] =0.0021; **(E)** ****p [plasma *vs.* WBF SMI 60 µm] <0.0001; *p [plasma *vs.* WBF SMI 4 µm] = 0.0193; ****p [WBF SMI 4 µm *vs*. WBF SMI 60 µm] <0.0001. **(F)** Calculated Pearson correlation of hu GM-CSF and interferon-gamma (IFNγ) secretion, analyzed by multiplex immunoassay, with % of TH1 in both populations was highly significant in WBF around SMI 60 µm but not SMI 4 µm. **(G)** Pearson correlation of IL17 secretion with % of TH17 in both populations was higher in WBF around SMI 60 µm compared to SMI 4 µm. Pearson r and p-value for SMI 4 µm and SMI 60 µm were denoted above the corresponding panel. The level for statistical significance was set at ^ns^p>0.05, *p<0.05, **p<0.002, ***p<0.0002, and ****p<0.0001, for all statistical tests (inter- and intra-individual comparison; n=7).

The immediate wound environment post-SMI implantation exhibited a complex yet distinct inflammasome contingent on SMI surface topography. To further understand immune cell populations and response towards exclusive surface antigens, immune profiling was conducted. Flow cytometric analysis showed no discernible effect of surface roughness on immune cell populations (**Figures 4A, B**; **Supplementary Figures S5, S6I**). CD4^+^ T cells, particularly TH1 and TH17, CM and EM subpopulations, were the main T cell subpopulations around both devices (**Figure 4C**; **Supplementary Figures S5, S6I**). Cytokine secretion analysis indicated a TH1 response through significant IFNγ, IL1b, and TNFα increase around both devices, with no surface topography influence on immune cell response (**Supplementary Figure S6A–H**). Gene expression analysis, however, revealed proinflammatory *IFNγ* increase (**Figure 4D**) and profibrotic marker *IL17* elevation (**Figure 4E**) around SMI 60 µm. Correlation analysis found a significant positive correlation between IL17A secretion and % of TH17 cells, as well as huGM-CSF and IFNγ expression with % of TH1 cells in SMI 60 µm-enclosed wound (**Figures 4F, G**). In contrast, no correlation between cytokine expression and TH1/TH17 immune cell profile was noted in acute SMI 4 µm wounds.

In essence, these findings confirm a predominant proinflammatory CD4^+^ TH1 and profibrotic TH17 response to SMI. This response remains consistent across diverse implant surface topographies. Silicone surface topographies do not influence T-cell proliferation or distribution of T-cell subpopulations; however, reducing surface roughness to Ra 4 µm mitigates proinflammatory and profibrotic immune cell responses.

### 3.4 Proinflammatory and profibrotic protein adhesion is amplified by rougher implant surface

**Figure 5:**
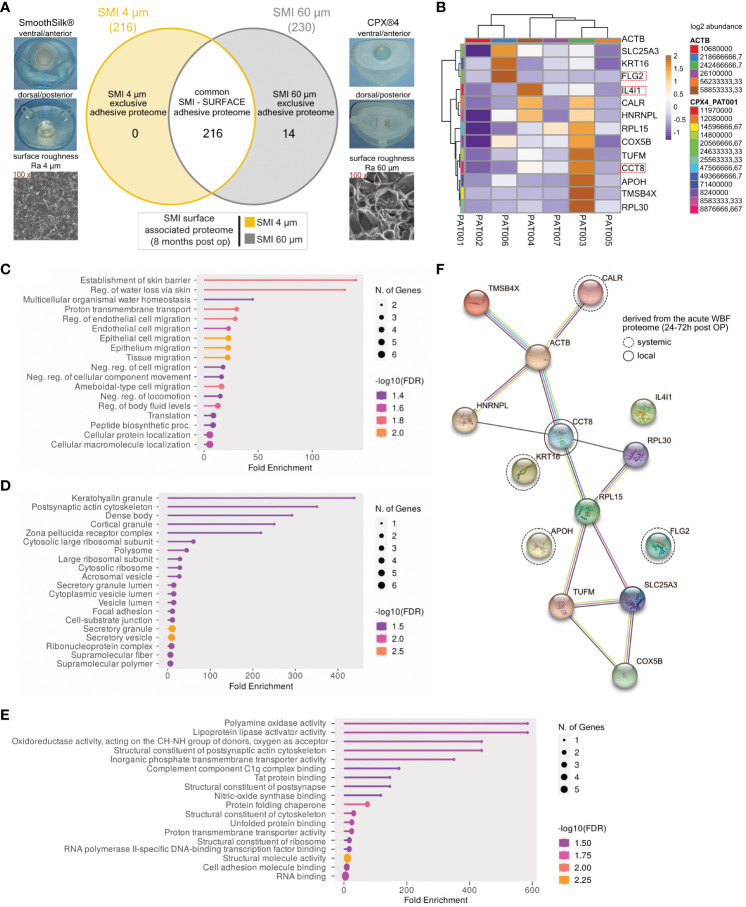
SMI topography affects surface-associated adhesive proteome. **(A)** Photo and SEM image of SMI 4 µm and SMI 60 µm and Venn diagram showing the distribution of proteins associated at silicone tissue expander surface, of both SMI 4 µm and SMI 60 µm (6 – 8 months post-op; n=7). Shaded yellow panel: SMI 4 µm surface exclusive adhesive proteome. Shaded grey panel: SMI 60 µm surface exclusive adhesive proteome. Clear panel: common adhesive proteome to both SMI. **(B)** Heatmap analysis of mean protein log2 transformed abundance of SMI 60 µm surface exclusive adhesive proteome. *Rows:* Clustered by Manhattan distance, average method, and tightest cluster first tree ordering. Columns: Clustered by correlation distance, average method, and tightest cluster first tree ordering. **(C)** GO biological process enrichment, **(D)** GO molecular function enrichment, **(E)** GO cellular component enrichment **(F)** protein-protein interaction regulatory network by K-means clustering based on STRING database, analysis of SMI 60 µm surface exclusive adhesive proteome (inter- and intra-individual comparison; n=7).

Our previous work elucidated the three-dimensional composition of the surface-associated proteome of SMIs, encompassing adhered plasma, local tissue-derived proteins in the acute wound, and those expressed in early fibrosis stages (13). This study hones in on the surface topography’s impact on protein adhesion post-breast implantation, focusing on the surface-exclusive proteomes. Interestingly, no specific protein adhesion to SMI 4 µm was found in all seven patients (**Figure 5A**). Among 14 proteins exclusive to the rougher surface (SMI 60 µm; **Supplementary Table S7**), we identified the T cell response enhancer CCT8, fibroblast growth factor receptor FLG, and M2 macrophage polarization marker IL4I1 (**Figure 5B**).

To comprehend the functions and fibrotic pathways of the exclusive adhesive proteome, we conducted GO biological process (**Figure 5C**), molecular function (**Figure 5D**), and cellular component enrichment analyses (**Figure 5E**), along with STRING PPI analysis (**Figure 5F**). GO analysis of SMI 60 µm exclusive proteome revealed associations with skin barrier establishment, cell migration biological processes, molecular roles like keratohyalin granule and secretory mechanisms, and cellular components including C1q complex binding and oxidases. We identified 3 plasma-derived and 11 local wound tissue-produced proteins in the acute wound (**Figure 5F**). STRING analysis yielded a PPI network predominantly expressed by local acute wound tissue, mainly in keratinocytes and lymphoblasts, with 14 nodes, 15 edges, average node degree 2.14, local clustering coefficient 0.619, expected edge number 8, and PPI enrichment p-value of 0.024 (**Figure 5F**).

These findings suggest escalated T cell response, fibrogenesis, and M2 macrophage activity due to increased SMI surface roughness, confirming heightened foreign body response to SMI 60 µm both in acute wounds and early-stage fibrosis 6 to 8 months post-implantation.

### 3.5 Enhanced implant encapsulation due to rougher surface texture

**Figure 6:**
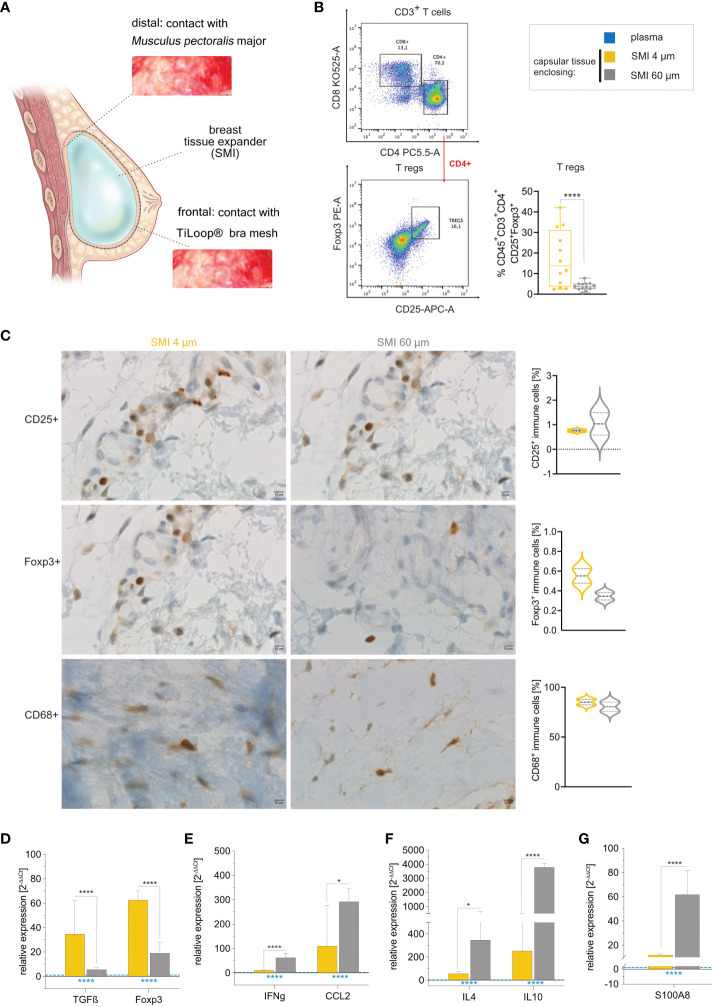
Reduction of SMI surface roughness from 60 to 4 µm tones down the FBR 6-8 months post-implantation. **(A)** Schematic representation of capsular tissue sample collection 6-8 months post-op at two different locations, frontal contact zone with the ® Bra mesh and distal contact zone with *M. pectoralis major.* **(B)** Flow cytometry revealed a significantly higher intracapsular CD4+ Treg population in capsular tissue formed around SMI 60 µm compared to SMI 4 µm. **(C)** Immunohistochemical staining of capsular tissue samples, harvested from encapsulated SMI 4 µm and SMI 60 µm for the Treg marker CD25 (panel 1) and Foxp3^+^ protein (panel 2) as well as macrophage marker CD68 protein (panel 3). Immune cells (brown) and negative stroma cells (blue). 100x magnification (scale 10 µm). **(D–G)** Intracapsular gene expression of **(D)** Treg (*TGFß* and *Foxp3) -* **(E)** M1 macrophage (*IFNγ* and *CCL2) -* and **(F)** M2 macrophage (*IL4* and *IL10*) – regulators and **(G)** fibrosis driver S1008 was evaluated by qPCR analysis. Unpaired t-test by two-stage linear step-up procedure of Benjamini, Krieger and Yekutieli: **(D)** *p[TGFß] = 0.016155; ****p[Foxp3] <0.000001, **(F)** ****p[IFNγ] = 0.000007; *p[CCL2] = 0.0182274, **(G)** *p[IL4] = 0.01723; ****p[IL10] <0.000001 and **(I)** ****p[S100A8] = 0.000022. The level for statistical significance was set at ^ns^p>0.05, *p<0.05, **p<0.002, ***p<0.0002, and ****p<0.0001, for all statistical tests (inter- and intra-individual comparison; n=7).

Our data aligns with our previous observation of a notably thicker capsule around the rougher device compared to SMI 4 µm (26). To probe implant surface roughness effects on capsular composition, we conducted immune profiling of intracapsular immune cells through flow cytometry (**Supplementary Figure S7**) and immunohistology on capsules harvested bilaterally 6-8 months post-implantation (**Figure 6A**). Flow cytometry revealed significant upregulation of intracapsular T regulatory cells (**Figure 6B**). This was further substantiated by immunohistology that showcased the enrichment of CD25+ immune cells with high inter-individual variance around the rougher device (**Figure 6C**, panel 1) and Foxp3+ immune cells around SMI 4 µm (**Figure 6C**, panel 2), and supported by increased *TGFβ* and *Foxp3* gene expression in tissue encapsulating SMI 4 µm (**Figure 6D**).

Initial mass spectrometric analysis of the acute wound and surface-adhesive proteome indicated differential macrophage polarization between the two devices (surface topographies). While CD68+ populations showed no significant differences regarding surface roughness (**Figure 6C**, panel 3). However, gene expression analysis revealed significantly increased intracapsular gene expression of macrophage M1 markers *IFNγ*, *CCL2* and subpopulation M2 markers *IL4*, and *IL10* around SMI 60 µm (**Figures 6E, F**). The augmented profibrotic response was confirmed by significantly elevated *S100A8* expression within the encapsulation of the rougher surface (**Figure 6G**).

### 3.6 Reduced implant surface roughness mitigates stress-induced t cell response in pericapsular inflammation

Heat shock proteins (HSPs) are stress-responsive molecules implicated in diverse pathophysiological processes. We previously identified HSP27, HSP70, and HSP60 in both acute wound and surface adhesive proteomes common to both implant surfaces (13). HSP60 accumulation in HSP60+ macrophages and fibroblasts at the capsule/implant interface illustrated its stress-driven role (14). Given HSP60’s ability to mirror stress on the implant and capsule, our investigation aimed to establish a correlation between intracapsular HSP60 levels and implant topography.

**Figure 7:**
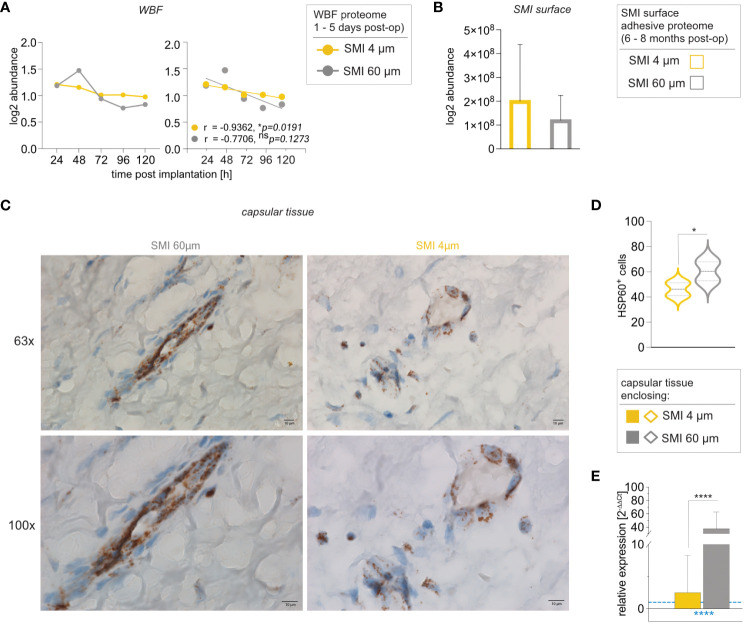
Reduction of SMI surface roughness from 60 to 4 µm decreases intracapsular HSP60 expression. Comparative analysis of **(A)** local tissue-derived HSPD1 log2 abundance (Left; dots with connecting lines, median shown) with calculated Pearson r of protein abundance x time point post-op (Right; scatterplot with simple regression line; Pearson r and p-value for both SMI were denoted above the corresponding panel): in WBF formed around both SMI 24 – 120 h post-implantation, **(B)** SMI surface-associated HSPD1 log2 abundance around both devices and **(C, D)** Immunohistochemical staining for HSP60 in capsular tissue samples, harvested from encapsulated SMI 4 µm and SMI 60 µm 6-8 months post-implantation (n=5 x two locations of sample isolation). **(C)** Cells expressing HSP60 antigenic determinants are stained brown and negative stroma cells are blue. Horizontal panel 1: 63x magnification (scale 10 µm) and panel 2: 100x magnification (scale 10 µm) **(D)** Statistical analysis of cells expressing HSP60 antigenic determinants: 2-way ANOVA of interaction (topography x location of sample isolation) main effect: F (1, 14) = 0.1763, ^ns^p = 0.6810; topography main effect: F(1,14) = 5.777, *p =0.0307; location of sample isolation main effect: F(1,14) = 4.508, ^ns^p = 0.0521. (inter- and intra-individual comparison; n=5; two locations of sample isolation) **(E)** Intracapsular *HSP60* gene expression in capsular tissue samples, harvested from encapsulated SMI 4 µm and SMI 60 µm 6-8 months post-implantation. Unpaired t-test by a two-stage linear step-up procedure of Benjamini, Krieger, and Yekutieli: **p[HSP60] = 0.003143. The level for statistical significance was set at ^ns^p ^>^0.05, *p<0.05, **p<0.002, ***p<0.0002, and ****p<0.0001, for all statistical tests (inter- and intra-individual comparison; n=7).

Notably, HSP60 protein abundance in wound-adjacent fibrous tissue around SMI 4 µm displayed a regressive correlation with time (**Figure 7A**), unlike SMI 60 µm (**Figure 7A**). HSP60 adhesion was evident on both surfaces 6 to 8 months post-op (**Figure 7B**). Remarkably, intracapsular *HSP60* gene expression was significantly higher (40x) in capsules formed on SMI 60 µm than on SMI 4 µm (**Figure 7C**). Immunohistochemical analysis supported this, revealing heightened tissue damage stress around SMI 60 µm (**Figure 7D**), resulting in more HSP60+ cells in the encapsulating tissue around SMI 60 µm.

These findings collectively suggest that reducing implant surface roughness significantly lessens tissue damage and capsular inflammation by mitigating pro-inflammatory mechanical stress mechanisms and impeding HSP60-mediated T-cell activation.

## 4 Discussion

During acute wound healing, a coordinated response involving hemostasis, immune cell recruitment, angiogenesis, and re-epithelialization is crucial (5, 10). Following implantation, fluid accumulation at the incision site triggers an immediate burst of systemic foreign body response (FBR), which decreases over the first days (13). Local tissue also contributes to the proinflammatory response over the short- and long-term, forming an adhesive and resident inflammatory matrix on the silicone implant (SMI) surfaces (13). Biomaterial surface properties, such as chemistry, mechanical characteristics, and topography, influence immune reactions (25, 28, 46). Our data show a gradual decrease in acute wound proteomic response around both SMIs, with distinct temporal sequences due to surface differences (**Figures 2C–J**; **Supplementary Figures S1, S2**). Implants with an average roughness of 4 μm can delay the initial fibrosis-inducing host-defense mechanisms, particularly by later accumulation of S100A proteins, which play roles in inflammation initiation and maintenance (47–50). Decreasing the SMI surface roughness to 4 µm not only slows the accumulation of certain S100 isoforms (A4, A6, A8) but also alters the expression of others (A7, A9, A10) (**Supplementary Figure S1I**). Moreover, the discovery of delayed TNFα activation in proximity to a 4µm Ra SMI, leading to a correspondingly shifted initiation of COL18A1 expression on the fourth day following implantation, aligns well with the concept of implant surface topography serving as a precision mechanism for regulating the initial inflammatory response and proinflammatory signaling following SMI placement.

Significantly, we observe an increased accumulation of fibrinogen alpha subunit (FGA; **Supplementary Figure 1D**) around the smoother SMI (Ra 4 µm), indicating heightened blood clot formation and potential infection protection (51–53). FGA also stabilizes wounds during early repair (54).

Reducing surface roughness from 60 to 4 µm notably affected wound proteins. Fibrosis progression-associated proteins (COL5A2, MMP19, S100A2; **Figures 3C, D**) were produced only around Ra 60 µm (**Figure 3E**; **Supplementary Figure S4**, while fibrosis resolution-related COL4A3 accumulated exclusively around Ra 4 µm implants (**Figure 3H**; **Supplementary Figure S3**). Furthermore, SMI surface topography impacts ECM turnover, evident in the COL 1/III and TIMP2/MMP2 stoichiometric ratio, indicating less pro-fibrotic reactivity around Ra 4 µm implants. Conversely, the rougher WBF around SMI (Ra 60 µm) exhibits higher levels of COL I and TIMP2, reflecting impaired wound healing/fibrosis (55–57).

The wound proteome formed solely around the rougher device reflected an upregulation of NFKβ signaling (GO enrichment analysis). NFKβ, crucial for immune cells and inflammation, likely contributes to chronic inflammation and implant-related issues (58, 59).

Prior findings highlighted neutrophil, granulocyte, and monocyte involvement in tissue response to both SMIs (13), indicating their role in the initial inflammatory storm. Macrophage response was consistent (**Supplementary Figure 1B**), but IFIT3 (**Supplementary Figure 1C**), a neutrophil degranulation activator (60, 61), was significantly enriched around the rougher device. Neutrophil granules, central in acute inflammation, contain activators for innate immunity components, promoting fibrosis, and enzymes for targeted tissue remodeling during fibrosis (62).

Effective healing typically involves a dominant T helper 1 (Th1) cell response, while chronic inflammation and potential fibrosis are linked to a prevalent T helper 2 (Th2) response and increased T helper 17 (Th17) cell presence (5, 10). Notably, T cell response can be influenced by heightened neutrophil degranulation, as neutrophil-released content enhances T cell activation, proliferation, and differentiation into TH1, TH17, and effector CD8+ T cells, promoting adaptive immune responses at inflammation sites (63–65).

Our findings reveal that implantation of SMI with Ra 60 µm triggers enhanced TH1 and TH17 cell recruitment. This also corresponds to significantly elevated gene expression of both pro-inflammatory (*IFNγ*) and pro-fibrotic (*IL17*) cytokines in the acute wound, particularly 72 hours post-operation (**Figure 4**). These results strongly indicate the impact of implant surface topography on early inflammatory response and fibrotic reactions. Importantly, our evidence demonstrates that reducing SMI surface topography to Ra 4 µm hinders SMI immunoreactivity, even within the initial five days following implantation.

Protein adherence to SMI surfaces reinforces silicone immunoreactivity in surrounding tissues (9, 12, 66). Protein adherence contributes to chronic local inflammation and fibrosis (67). Our research identified biomarkers associated with both silicone surfaces in acute wounds and even after 8 months, indicating prolonged pro-inflammatory and pro-fibrotic conditions (13). Strikingly, we found unique protein adhesion on the rougher SMI, enriching pathogenic early-stage fibrosis markers (**Figure 5**). Interestingly, among the 14 associated proteins, we found the pro-inflammatory driver FLG2 (68, 69), along with IL-4(II) that promotes pro-fibrotic macrophage activation and M2 polarization (70) as well as CCT8, a regulator of T cell activation and TH1 polarization. FLG2 and CCT8 were identified among the common acute wound bed proteomes around both devices (13) and, remarkably, the enrichment of these three factors directly corresponds to an exacerbation of early-stage fibrosis, evident exclusively in the SMI surface with a Ra of 60 µm, observed 8 months after implantation.

Our prior *in vitro* studies demonstrated silicone’s immunoreactivity, sparking a specific immune response in capsular tissue involving macrophages, T cells, and DCs (14). In a mouse model, using miniaturized implants with an average roughness of 4 μm yielded the least capsular thickness; however, this effect was absent in T cell-deficient mice (25). This suggests that T cells, particularly intracapsular Tregs and TH17 cells, play a crucial role in implant encapsulation (15). Notably, our earlier research confirmed reduced implant encapsulation around 4 μm roughness implants in human patients (26). Further investigation of the capsule’s composition highlighted increased intracapsular Tregs, along with elevated gene expression of *Foxp3* and *TGFβ* around SMI with an average roughness of 4 µm, without impacting T helper cell subpopulations (**Figure 6**). Furthermore, capsules formed around rougher devices with an average roughness of Ra 60 µm exhibited significantly higher expression of *IFNγ*, *CCL2*, *IL4*, and *IL10* (**Figure 6**). This indicates enhanced macrophage activation and M2 polarization. The thicker pseudo synovium and increased S1008 expression (**Figure 6**) also underscored the heightened foreign body response (FBR) to the rougher implant.

Lastly, our discovery involves intracapsular expression of HSP60 (**Figure 7**). The placement of a breast implant triggers a host response to reactive oxygen species (ROS), nitric oxide, and mechanical stress, which initiates tissue destruction (5, 10). We previously found HSP60 to be involved in the inflammatory response at the wound site and attached to SMI surfaces eight months post-operation (13). This stress-induced protein contributes to inflammation persistence by prompting secretion of proinflammatory cytokine IFN-γ (71), activating bacterial HSP-responsive gamma delta T cells, and becoming a target for autoreactive HSP60-specific T-cell responses, significantly disrupting wound healing (72–75).

Notably, textured implant surfaces tend to become smoother over time due to shearing, and mechanical shear stress can incite pronounced inflammatory responses (76), possibly contributing to double capsule formation (77). As expected, our data demonstrates significantly increased intracapsular expression of HSP60 around the rougher encapsulated device (SMI 60 µm) after eight months (**Figure 7**). This highlights the immunomodulatory influence of SMI surface topography on both the response to tissue damage and the process of tissue repair during wound healing. The restricted use of patient samples in a clinical trial could account for another limitation of our study. Testing HSP60-mediated T-cell activation, of both peripheral and intracapsular T cells in all seven patients, was beyond the scope of this paper and underlines the difficulty of collecting data on human patients.

**Figure 8:**
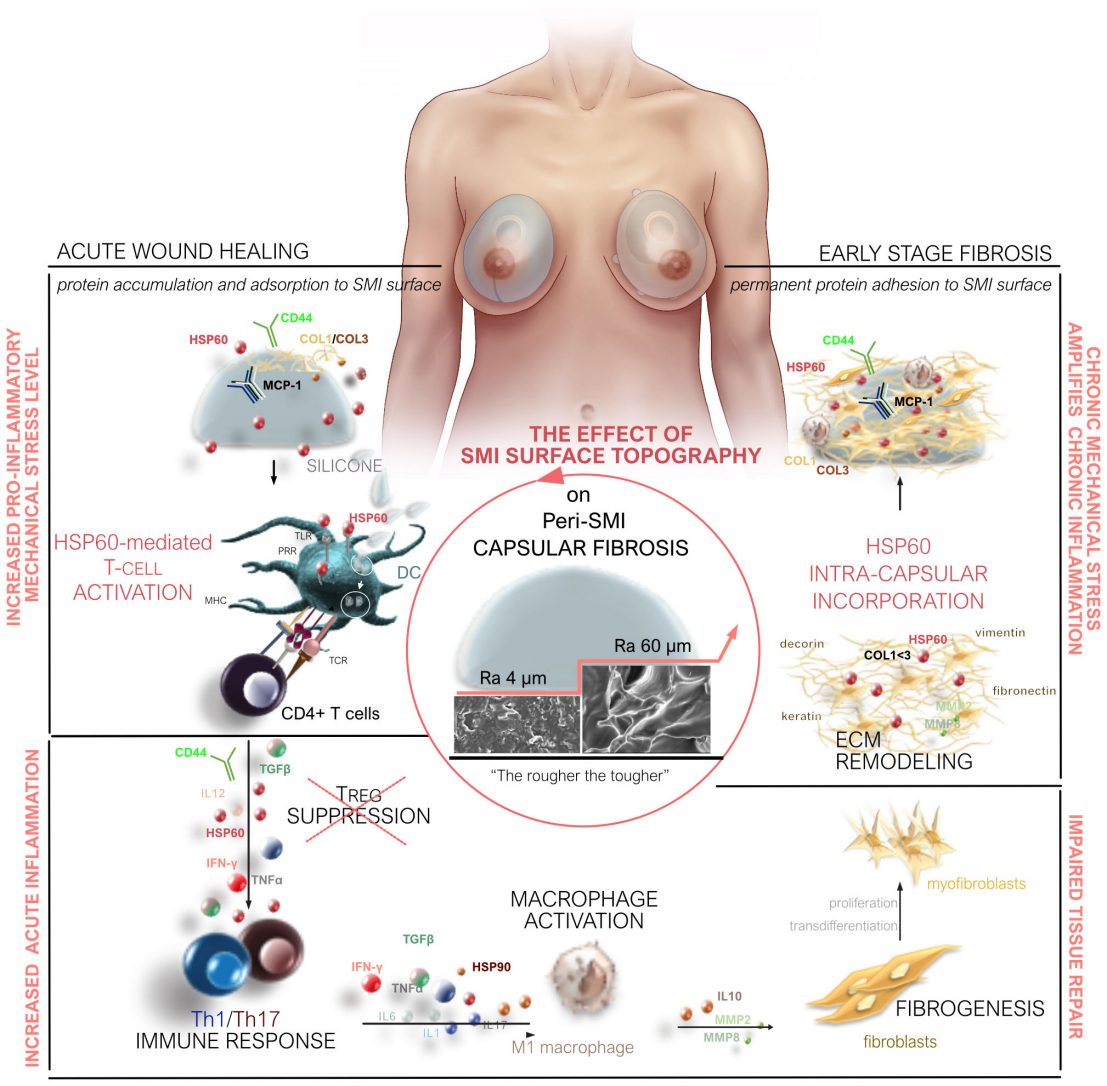
Microtopography Effects on Capsular Fibrosis. Microtopography significantly impacts capsular fibrosis through heightened pro-inflammatory mechanical stress on rougher SMI surfaces (Ra 60 µm). This stress enhances cell adhesion-mediated inflammatory signaling, intensifying the inflammatory response. Rougher surfaces promote a proinflammatory immune cell phenotype, with HSP-60-mediated T-cell activation contributing to increased inflammation during implantation. The elevated cell adhesion on rougher surfaces, compared to smoother ones, potentially heightens the activation of immune cells, particularly macrophages. Proteomic analysis of rougher implant surfaces reveals a distinctive biomolecular interaction pattern, altering protein adsorption and potentially amplifying downstream signaling, prolonging inflammation. The varied inflammatory milieu around implants with different surface roughness levels suggests an impact on cytokine and chemokine expression, potentially leading to an imbalanced cytokine profile. Long-term chronic inflammation impairs tissue repair by promoting excessive fibrogenesis.

Our study provides a comprehensive understanding of capsular fibrosis following simultaneous prophylactic NSME and breast-tissue expander-based reconstruction, comparing two SMI variants (Ra 4 and 60 µm). Surface topography significantly impacts acute and chronic responses, shaping the acute wound proteome and early-stage fibrotic capsular tissue (**Figure 8**). Increased pro-inflammatory, pro-fibrotic activation, TH1/TH17 immune cell responses, and ECM turnover are seen in the rougher device (Ra 60 µm) directly post-op. Moreover, an exclusive proteome adheres to this rougher SMI, further indicating pro-fibrotic modulation and increased immune response. The cumulative result is a thicker capsule and pronounced pseudo-synovium development adjacent to the silicone surface.

A significant discovery has been made regarding the distinctive three-dimensional composition of a pro-fibrotic SMI surface-associated/adhesive proteome. This discovery involves the identification of two key observations: (i) the accumulation of plasma-derived proteins in the immediate wound vicinity around both SMI devices and (ii) the specific adhesion of these components to the surface roughness of the devices, with subsequent expression inside the capsule during the early stages of fibrosis development. Notably, among these observations, we have pinpointed potential long-term capsular fibrosis markers, specifically S100A8 and FLG2, alongside CTC8, which exhibits a distinct “signature” related to the adhesion of plasma proteins to various silicone types. This breakthrough offers innovative diagnostic targets for the long-term monitoring of capsular fibrosis and the possibility of capsular contracture. Currently, we are in the process of investigating the potential utility of these candidates in an ELISA-based testing system, known as SILISA^®^ (12), to assess the risk of fibrosis development directly in the serum following SMI implantation.

Altogether, this study demonstrates that surface topography acts as a master regulator, orchestrating immune reactions and influencing wound healing trajectories. We observed a notable decrease in inflammation with smoother surface microtopography (Ra 4 µm). While the data robustly support this observation, elucidating the underlying mechanisms is crucial for a thorough interpretation of our findings.

Surface roughness is known to influence cell-material interactions, prompting us to consider potential mechanisms that may explain the observed immunomodulatory effects. One plausible explanation is related to reduced cell adhesion on smoother surfaces. Enhanced surface smoothness could lead to decreased cell adhesion, particularly of immune cells like macrophages, potentially resulting in a less pronounced activation of immune signaling pathways.

The unique proteomic signature associated with the smoother implant surface suggests differential biomolecular interactions. Altered protein adsorption patterns may impact downstream signaling cascades, influencing the immune response. Investigating the specific proteins adhering to different surface topographies could offer insights into the molecular basis of the observed immunomodulation.

Surface microtopography has been shown to influence immune cell behavior and phenotype. Smoother surfaces may promote a more anti-inflammatory or regulatory phenotype in immune cells, contributing to a dampened proinflammatory response. Understanding the surface-induced modulation of immune cell polarization could provide mechanistic insights.

The altered inflammatory milieu observed around implants with different surface roughness levels suggests a potential impact on cytokine and chemokine expression. Smoother surfaces may attenuate the release of proinflammatory mediators and promote a more balanced cytokine profile, contributing to reduced inflammation.

While these proposed mechanisms provide a conceptual framework, we acknowledge the need for further in-depth studies, including molecular and cellular investigations, to unravel the precise molecular pathways involved. Our study serves as a foundational exploration, and we are committed to conducting additional mechanistic investigations to enhance our understanding of the interplay between silicone implant surface microtopography and inflammatory responses.

As medical advancements continue, harnessing this understanding could pave the way for tailored implant designs that optimize immune interactions, enhance tissue integration, and ultimately improve patient outcomes.

## 5 Future perspectives

### 5.1 Novelty and implications

This study presents a comprehensive exploration of the impact of surface topography on acute and chronic responses following breast implantation, shedding light on the intricate interplay between implant properties and the host immune system. Our findings reveal the distinct proteomic signatures associated with different surface roughness levels, providing novel insights into the mechanisms underlying proinflammatory and profibrotic responses. The identification of specific proteins adhering to implant surfaces and their correlation with early-stage fibrosis markers opens avenues for potential diagnostic targets and monitoring strategies.

### 5.2 Shortcomings and areas for further investigation

Despite the robustness of our study, certain limitations warrant acknowledgment. The use of patient samples in a clinical trial imposes constraints on sample size, necessitating larger cohorts for validation and generalization of findings. The investigation of HSP60-mediated T-cell activation in both peripheral and intracapsular T cells, while acknowledged as a potential contributor to inflammation, lies beyond the scope of this paper and calls for dedicated research. Furthermore, while our study primarily focuses on acute and early-stage responses, the long-term implications of surface topography on implant performance and patient outcomes remain an essential avenue for future research.

### 5.3 Clinical translation and therapeutic implications

The identified proteomic signatures associated with different surface topographies pave the way for the development of precision diagnostics and monitoring tools. The SILISA^®^ system, currently under investigation, holds promise as a potential method for assessing the risk of fibrosis development following SMI implantation. The understanding that surface topography acts as a master regulator opens avenues for designing implants tailored to optimize immune interactions, improve tissue integration, and mitigate complications such as capsular contracture. These insights have implications not only in breast reconstruction but also in diverse fields of implantology, encouraging the development of implants with enhanced biocompatibility.

### 5.4 Tailoring implant designs for optimized patient outcomes

As medical advancements progress, the integration of this understanding into the design of future implants could revolutionize patient care. Tailoring implants based on surface topography to modulate immune reactions and influence wound healing trajectories may lead to improved outcomes and reduced complications. The potential to mitigate proinflammatory and profibrotic responses by altering surface roughness opens avenues for personalized medicine in implantology, emphasizing the importance of considering individual patient profiles for optimized results.

In conclusion, this study not only expands our knowledge of the intricate dynamics between implant surface properties and host responses but also lays the foundation for translational applications that could redefine standards in implant design and patient care. The identified proteomic signatures, diagnostic targets, and therapeutic implications contribute to a growing body of evidence that aims to enhance the safety and efficacy of implant-based procedures, ultimately benefiting the well-being of patients undergoing such interventions.

## Data availability statement

The datasets presented in this study can be found in online repositories. The names of the repository/repositories and accession number(s) can be found below: http://www.proteomexchange.org/, PXD039840.

## Ethics statement

The studies involving humans were approved by Institutional Ethical Committee of the Medical University Innsbruck, Austria (protocol code 1325/2019, 23 January 2020) and the Austrian Federal Office for Safety in Health Care (approval number; 13340962). The studies were conducted in accordance with the local legislation and institutional requirements. The participants provided their written informed consent to participate in this study. Written informed consent was obtained from the individual(s) for the publication of any potentially identifiable images or data included in this article.

## Author contributions

IS: Conceptualization, Data curation, Formal analysis, Investigation, Methodology, Project administration, Resources, Software, Supervision, Validation, Visualization, Writing – original draft, Writing – review & editing. KF: Data curation, Methodology, Validation, Writing – review & editing. CT: Methodology, Writing – review & editing. EP: Methodology, Investigation, Writing – review & editing. SS: Data curation, Project administration, Validation, Writing – review & editing.. BZ: Methodology, Writing – review & editing. AB: Methodology, Writing – review & editing. NH-K: Methodology, Writing – review & editing. LB: Methodology, Writing – review & editing. TS: Methodology, Visualization, Writing – review & editing. BS: Methodology, Writing – review & editing. DE: Methodology, Writing – review & editing. CB: Methodology, Writing – review & editing. DW: Conceptualization, Funding acquisition, Investigation, Methodology, Supervision, Validation, Writing – review & editing.
