# Supplementary material for: Silicone implant surface microtopography modulates inflammation and tissue repair in capsular fibrosis
Source: Front Immunol. 2024 Mar 19;15:1342895. doi: 10.3389/fimmu.2024.1342895 (PMC10985323; doi:10.3389/fimmu.2024.1342895)
Supplement: Supplementary file 1 [file DataSheet_1.docx]

Supplementary Material

# Supplementary Data

## Supplementary Figures

**Supplementary Figure 1.** SMI surface roughness reduction effect on common plasma-derived wound bed fluid proteome expression formed intra-individually around SMI 4 µm and SMI 60 µm post-implantation (24 – 120 h post-op; n=7). **(A)** Heatmap analysis of DEP log2 abundance in the plasma-derived wound proteome. Rows: no clustering. Columns: Columns are centered; unit variance scaling is applied to columns. Columns are clustered using correlation distance and average linkage. (**B-J)** Comparative analysis of protein abundance over the first 120 h post-op: involved in mechanisms of (**B)** macrophage recruitment and activation, **(C)** neutrophil inflammatory response, **(D)** fibrogenesis, as **(E)** collagenous ECM components, **(G)** enzymes of ECM turn-over as well as members of the fibrosis driving **(I)** S100A family. Pearson r and p-value for SMI 4 µm and SMI 60 µm were denoted above the corresponding panel. Time-course log2abundance ratio of **(F)** COLI/COLII, **(H)** TIMP2/MMP2, and **(J)** S100A8/A9 protein ration around both SMI (24 – 120 h post-op). Paired t-test comparison significances are denoted in the graph. The level for statistical significance was set at ^ns^p >0.05, *p<0.05, **p<0.002, ***p<0.0002, and ****p<0.0001, for all statistical tests (inter- and intra-individual comparison; n=7).

**Supplementary Figure 2.** SMI surface roughness reduction effect on common local wound bed fluid proteome formed intra-individually around SMI 4 µm and SMI 60 µm post-implantation 1 to 5 days post-surgery (24 – 120 h post-op; n=7). **(A)** Heatmap analysis of DEP log2 abundance in the local-derived wound proteome. Rows: no clustering. Columns: Columns are centered; unit variance scaling is applied to columns. Columns are clustered using correlation distance and average linkage. (**B-H)** Comparative analysis of protein abundance over the first 120 h post-op: involved (**B)** Interferon-induced proteins, **(C)** latent TGFβ binding proteins, **(D)** member of TNF receptor family, **(E)** subunits of NFκB, **(F)** proteins involved in macrophage activation and polarization, **(G)** collagens and members of proinflammatory mediator **(H)** S100A family. Pearson r and p-value for SMI 4 µm and SMI 60 µm were denoted above the corresponding panel. The level for statistical significance was set at ^ns^p >0.05, *p<0.05, **p<0.002, ***p<0.0002, and ****p<0.0001, for all statistical tests (inter- and intra-individual comparison; n=7).

**Supplementary Figure 3.** SMI 4 µm exclusive wound proteome 24 – 120 h post-op. (**A)** functional categorization in the context of tissue repair of SMI 4 µm exclusive wound proteome (24 – 120 h post-op; n=7. Heatmap analysis of DEP in the **(B)** plasma-derived and **(C)** local wound proteome formed around SMI 4 µm. *Rows:* Clustered by Manhattan distance, average method, and tightest cluster first tree ordering. Columns: Clustered by correlation distance, average method, and tightest cluster first tree ordering. The level for statistical significance was set at ^ns^p >0.05, *p<0.05, **p<0.002, ***p<0.0002, and ****p<0.0001, for all statistical tests (inter- and intra-individual comparison; n=7).

 **Supplementary Figure 4.** SMI 60 µm exclusive wound proteome 24 – 120 h post-op. (**A)** functional categorization in the context of tissue repair of SMI 60 µm exclusive wound proteome (24 – 120 h post-op; n=7) **(b)** Heatmap analysis of DEP in the **(B)** plasma-derived and **(C)** local wound proteome formed around SMI 60 µm. *Rows:* Clustered by Manhattan distance, average method, and tightest cluster first tree ordering. Columns: Clustered by correlation distance, average method, and tightest cluster first tree ordering. The level for statistical significance was set at ^ns^p >0.05, *p<0.05, **p<0.002, ***p<0.0002, and ****p<0.0001, for all statistical tests (inter- and intra-individual comparison; n=7).

 **Supplemntary Figure 5.** Representative flow cytometry gating strategy for TH1/TH2 immunoprofiling in **(A)** plasma pre-OP, WBF formed around **(B)** SMI 60µm and **(C)** SMI 4 µm 24 h post-op (PAT001_004).

**Supplementary Figure 6.** Quantification of WBF **(A-H)** immune cell response on day 1 to 5 post-op and (i) CD4^+^ T cell sub-populations in plasma pre-OP, WBF formed around SMI 60 µm and SMI 4 µm 24 – 72 h post-op. Biological samples were evaluated by **(A-H)** immunoblot and **(I)** flow cytometry analysis. 2-way ANOVA **(A-I):** ^ns^p >0.05. The level for statistical significance was set at ^ns^p >0.05, *p<0.05, **p<0.002, ***p<0.0002, and ****p<0.0001, for all statistical tests (inter- and intra-individual comparison; n=7).

**Supplementary Figure 7.** Representative flow cytometry gating strategy for Tregs immunoprofiling in **(A)** plasma pre-OP, and capsular tissue formed around **(B)** SMI 60µm and **(C)** SMI 4 µm 6 – 8 months post-SMI-implantation (PAT 001_004).

**Supplementary Figure 8.** Immunohistochemical staining of capsular tissue from PAT001_001 for CD3, CD4, CD25, Foxp3 and CD68 immune cell marker (magnification: 20x; scale: 100 µm). Immune cells (purple) and stroma cells (green).

**Supplementary Figure 9.** Immunohistochemical staining of capsular tissue from PAT001_002 for CD3, CD4, CD25, Foxp3 and CD68 immune cell marker (magnification: 20x; scale: 100 µm). Immune cells (purple) and stroma cells (green).

**Supplementary Figure 10.** Immunohistochemical staining of capsular tissue from PAT001_003 for CD3, CD4, CD25, Foxp3 and CD68 immune cell marker (magnification: 20x; scale: 100 µm). Immune cells (purple) and stroma cells (green).

**Supplementary Figure 1.** Immunohistochemical staining of capsular tissue from PAT001_004 for CD3, CD4, CD25, Foxp3 and CD68 immune cell marker (magnification: 20x; scale: 100 µm). Immune cells (purple) and stroma cells (green).

**Supplementary Figure 12.** Immunohistochemical staining of capsular tissue from PAT001_005 for CD3, CD4, CD25, Foxp3 and CD68 immune cell marker (magnification: 20x; scale: 100 µm). Immune cells (purple) and stroma cells (green).

**Supplementary Figure 13.** Immunohistochemical staining of capsular tissue from PAT001_006 for CD3, CD4, CD25, Foxp3 and CD68 immune cell marker (magnification: 20x; scale: 100 µm). Immune cells (purple) and stroma cells (green).

**Supplementary Figure 14.** Immunohistochemical staining of capsular tissue from PAT001_007 for CD3, CD4, CD25, Foxp3 and CD68 immune cell marker(magnification: 20x; scale: 100 µm). Immune cells (purple) and stroma cells (green).

## Supplementary Tables

Supplementary Tables are provided as individual Excel data sheet .xls files.

**Supplementary Table 1.** Mass spectrometry log2 abundances of the identified common plasma-derived wound proteome in WBF 1-5 days post-op.

**Supplementary Table 2.** Mass spectrometry log2 abundances of the identified common local wound proteome in WBF 1-5 days post-op.

**Supplementary Table 3.** Mass spectrometry log2 abundances of the identified SMI 4 µm exclusive plasma-derived wound proteome in WBF 1-5 days post-op.

**Supplementary Table 4.** Mass spectrometry log2 abundances of the identified SMI 60 µm exclusive plasma-derived wound proteome in WBF 1-5 days post-op.

**Supplementary Table 5.** Mass spectrometry log2 abundances of the identified SMI 4 µm exclusive local wound proteome in WBF 1-5 days post-op.

**Supplementary Table 6**. Mass spectrometry log2 abundance results of the identified SMI 60 µm exclusive local wound proteome in WBF 1-5 days post-op.

**Supplementary Table 7.** Mass spectrometry log2 abundances of the identified total SMI 4 µm and 60 µm surface adhesive proteome 6-8 months post-op.

**Supplementary Table 8.** shows mass spectrometry log2 abundances of the identified SMI 60 µm surface-exclusive adhesive proteome 6-8 months post-op.
